# Acid-Sensing Ion Channels in Zebrafish

**DOI:** 10.3390/ani11082471

**Published:** 2021-08-23

**Authors:** Giuseppe Montalbano, Maria Levanti, Kamel Mhalhel, Francesco Abbate, Rosaria Laurà, Maria Cristina Guerrera, Marialuisa Aragona, Antonino Germanà

**Affiliations:** Zebrafish Neuromorphology Lab, Department of Veterinary Sciences, University of Messina, 98168 Messina, Italy; gmontalbano@unime.it (G.M.); kamel.mhalhel@unime.it (K.M.); abbatef@unime.it (F.A.); laurar@unime.it (R.L.); mguerrera@unime.it (M.C.G.); mlaragona@unime.it (M.A.); agermana@unime.it (A.G.)

**Keywords:** ASICs, ion channels, zebrafish, sensory organs, gills, gut, brain

## Abstract

**Simple Summary:**

The present review collects data regarding the presence of ASICs (acid-sensing ion channels) in zebrafish, which have become, over several years, an important experimental model for the study of various diseases. ASICs are a family of ion channels involved in the perception of different types of stimuli. They are excitatory receptors for extracellular H^+^ involved in synaptic transmission, the peripheral perception of pain and in chemical or mechanosensation.

**Abstract:**

The ASICs, in mammals as in fish, control deviations from the physiological values of extracellular pH, and are involved in mechanoreception, nociception, or taste receptions. They are widely expressed in the central and peripheral nervous system. In this review, we summarized the data about the presence and localization of ASICs in different organs of zebrafish that represent one of the most used experimental models for the study of several diseases. In particular, we analyzed the data obtained by immunohistochemical and molecular biology techniques concerning the presence and expression of ASICs in the sensory organs, such as the olfactory rosette, lateral line, inner ear, taste buds, and in the gut and brain of zebrafish.

## 1. Introduction

During the last decade, different types of ion channels, including acid-sensing ionic channels (ASICs), calcium-activated potassium (SK1), voltage-gated potassium channels (KV2), and transient receptor potential (TRP) family members, have been identified and investigated in many vertebrate and invertebrate species. Encoded by several genes in all cells type, ion channels show a wide diversity in molecular structure, selectivity to ions, and mechanisms of operation. However, they share the general structural feature of a pore that provides a pathway for charged ions to cross the plasma membrane down their electrochemical gradient. The present chapter covers the intriguing acid-sensing ionic channels in different zebrafish organs, highlighting their structures and function.

### 1.1. The Acid-Sensing Ion Channel Superfamily

**Figure 1:**
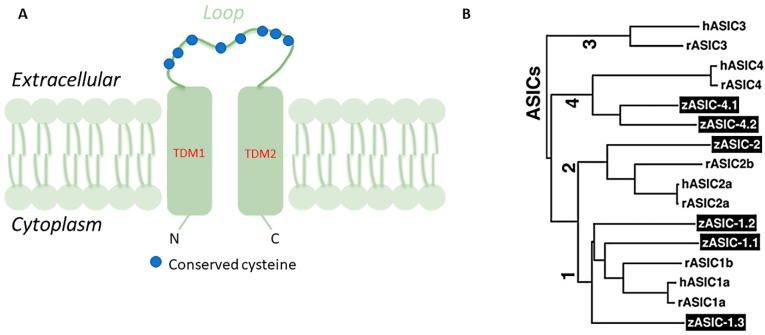
(**A**) Schematic view of one ASIC subunit: Each subunit has two hydrophobic transmembrane domains, a large cysteine-rich extracellular loop, and short intracellular N- and C- termini; (**B**) phylogenetic tree illustrating the relationship of zASICs and other ASICs from human (h) and rat (r) (Paukert et al., 2004 modified).

Acid-sensing ion channels (ASICs) are Na^+^ channels gated by extracellular H^+^ and are widely expressed in the mammalian central and peripheral nerve systems [1]. ASICs are part of the degenerin/epithelial sodium (Na) channel (DEG/ENaC) superfamily whose feature is high permeability to Na that could be blocked by amiloride. Structurally, ASICs consist of two hydrophobic transmembrane domains (TMD) of 20 amino acids approximately, TMD1 and TMD2, a large domain of around 370 amino acids forming an extracellular loop of 14 conserved cysteines, and a kind of short cytoplasmic amino and carboxyl termini of 35–90 amino acids (Figure 1A) [2,3,4]. Six ASIC proteins, encoded by four genes, have been identified as ASIC1a, ASIC1b, ASIC2a, ASIC2b, ASIC3, and ASIC4 in mammals [5]. Functionally, ASICs monitor moderate deviations from the physiological values of extracellular pH and also participate in mechanoreception and nociception [2,6,7,8,9] or taste receptors [10,11]. ASICs are homo- or hetero-oligomeric assemblies of individual subunits [12,13,14]. The *asic1a* subunit is widely expressed in the central and peripheral nervous systems and contributes to synaptic transmission [6,15,16]. *asic1b* and *asic3* are expressed in the peripheral nervous system where they would seem to be involved in pain perception [8,17,18,19]. The analgesic effects associated with the inhibition of those ASICs in animals suggest a role in pain. Particularly, in mice with targeted disruptions of the ASIC3 demonstrated the role of ASIC3 in modulating high-intensity stimuli perception, of great importance for the production of pain after tissue inflammation [8,17,19]. *asic2a* is extensively expressed in the brain and contributes to synaptic transmission [14,18]. *asic4* transcripts are abundantly distributed in the adult wild-type mouse brain [20], but in humans, *asic4* mRNA is expressed mainly in the pituitary gland while its expression in other parts of the brain is weak [21]. Moreover, ASICs are expressed in dorsal root ganglia (DRG), afferent gastrointestinal neurons [16] and in neurons of the myenteric and submucous plexuses [22]. 

### 1.2. ASICs in Zebrafish

Fish are, phylogenetically, the closest vertebrate group to amphibians and gave rise to mammals. Living in aquatic ecosystems, fish have different life histories and ecological pressures casting their evolution compared to mammals. An examination of the research findings from fish and mammals pointed up the extent of evolutionary conservation of ASICs across these animal taxa. Indeed, the proton-mediated activation of ASICs was highlighted in teleosts, while in a jawless fish and cartilaginous fish, ASICs were not activated by protons [23]. In addition, while in mammals the major routes for sodium chloride absorption are electroneutral absorption via the combined activity of electroneutral Na^+^/H^+^ exchangers and Cl−/HCO3− exchanger beside the electrogenic absorption via epithelial Na^+^ channels (ENaC) at the mucosal side of the intestinal epithelium, the Na^+^ uptake in the teleost was channelized by the ASIC family members [24]. ASIC, localized on the apical membranes of the ionocytes in FW trout gills, takes up Na^+^ from environmental FW in exchange for H^+^ through vacuolar-type H^+^-ATPase (VHA) [25]. In zebrafish, the involvement of the ASICs channels in Na^+^ uptake was proved [26].

The zebrafish is a model organism for studies in genetics and developmental biology that offers very advantageous features over other vertebrate models, including high fecundity, clear embryos, rapid development, straightforward analysis of gene expression, and detection of developmental abnormalities in mutant fish [27,28,29]. Similar to mammals, the zebrafish has six ASIC subunits denominated zASIC1.1, zASIC1.2, zASIC1.3, zASIC2, zASIC4.1, and zASIC4.2, encoded by six different genes. zASICs share the basic functional properties of mammalian ASICs: receptors of extracellular H^+^, Na^+^ selectivity, and the inhibiting effects of amiloride [30]. *zasic1.1*, *zasic2*, and *zasic4.1* are orthologs of mammalian *asic1*, *asic2*, and *asic4,* respectively, while *zasic1.2* and *zasic1.3* are paralogs of *zasic1.1* and, finally, *zasic4.2*, is a paralog of *zasic4.1* (Figure 1B) [30]. The proteins coded by *zasics* have molecular masses of around 60 kDa and share 60–75% of amino acid identity with rat and human ASICs [31]. Single ASIC subunits assemble to create functional homomeric or heteromeric ASICs with different surface expression levels on the plasma membrane and consequently different properties. A small region post-TMD has been found to be important in the gating mechanisms of channels in the *asic4* gene family [31]. ASICs have been demonstrated in zebrafish sensory organs, gut, gills, and brain using immunohistochemistry, molecular biology techniques, in situ hybridization, Western blot, and RT-PCR, where they ensure nerve transduction (Table 1). Additionally, as in rainbow trout, the ASICs involvement in sodium uptake has been demonstrated in adult zebrafish [31]. However, in larval zebrafish, the ASICs role in Na^+^ uptake has not been confirmed employing pharmacological and loss of function genetic approaches [32]. 

## 2. ASICs in Zebrafish Sensory Organs

Fish are able to transmit chemical or mechanical stimuli from the aquatic environment through a well-organized sensory system. The sensory organs in fish are represented by the olfactory rosette, lateral line, inner ear, taste buds, and chemosensory cells scattered throughout the epidermis and gills. ASICs have been observed in all the sensory organs of zebrafish, although with differences in the distribution between adult and larvae [33,34,35]. 

### 2.1. Lateral Line and Inner Ear

Neuromasts represent the sensory units of the lateral line system (LLS) of teleosts and are considered the morpho-functional unit of the LLS. In zebrafish, the neuromasts are grouped into superficial and deep neuromasts. They consist of centrally located sensory cells, supporting and mantle cells [36,37,38]. Lateral line ciliated sensory cells have their mechanosensitive stereocilia embedded in a gelatinous dome. The hair cells are involved in the perception of the water flow and movement [39]. They act as mechanoreceptors by converting mechanical stimuli into electrochemical signals [40]. The role of these cells is given by the presence on the sensory nerve endings and specialized sensory cells of mechanotransducer ion channels that generate a flow of ions in response to mechanical stimuli [7,41]. ASIC1 and ASIC3 immunoreactivity was found in the hair cells of neuromasts, while ASIC2 immunoreactivity was only observed in the nerves supplying the neuromasts. The nonsensory cells of the neuromasts, supporting and mantle cells, also displayed ASIC4 [42]. The inner ear consists of the labyrinth formed by three semicircular canals, the utricle, the vestibular organ, the sacculus, the organ of sound reception, and the lagena, the organ of orientation and hearing [43,44]. At the base of each semicircular canal, there is a dilated sac called a bony ampulla. Each ampulla has a sensory epithelium in the form of a round patch, the crista ampullaris [43,44]. The utricle, sacculus and lagena also have sensory cells termed macula, whose kinocilia and stereocilia are connected to the otoliths, dense calcareous structures [39,43,44]. ASIC1 and ASIC3 were observed in the inner ear neurosensory cells, while ASIC2 was demonstrated only in the nerves supplying them. The specific immunoreactivity for ASIC4 was not found [45]. 

### 2.2. Taste Buds 

Taste buds are chemosensory organs devoted to evaluating food composition and detecting variations in the environmental chemical composition [46]. They are distributed in the skin surface of the head, lips, and barbels and the intraoral cavity and oropharyngeal apparatus. The taste buds are intraepithelial sensory organs localized on a minor dermal papilla and are onion shaped. They are composed of modified epithelial sensory, supporting and basal cells; the fusiform-like sensory cells are arranged vertically within the papilla [46]. According to their electron density, sensory cells are classified into two main populations, the dark cells, having an apex with many short microvilli, and the light cells characterized by one single large microvillus at the apical border [46]. A third type of sensory cells present in zebrafish taste buds was identified by low electron density and a brush-like apex with many tiny microvilli [46]. The basal poles of the sensory cells are reached by nerve endings coming from the facial, glossopharyngeal, or vagal nerves [47]. *asic* mRNA and protein were detected in the taste buds of adult zebrafish. The anti-ASIC1 antibody identified, by Western blot analysis, a protein band with an estimated molecular weight of about 60 kDa, the anti-ASIC2 antibody detected a single protein band with an estimated molecular weight of about 64 kDa, and also the antibody against ASIC4 detected a single band with an estimated molecular weight of 64kDa [48]. Western blot analysis did not detect any ASIC3 protein. The immunohistochemical results were in agreement with the results obtained by Western blot analysis. ASIC2 and ASIC4 were localized in taste buds or in the nerves supplying them. ASIC4 was found in the oral and cutaneous taste buds. ASIC1 and ASIC3 positive immunostaining was not detected in the taste buds of adult zebrafish. In most taste buds, ASIC4 was found in all cells, but, in some cases, ASIC4 was demonstrated only in a calretinin-expressing cell subtype [48,49]. 

### 2.3. Olfactory Epithelium

The olfactory organ consists of numerous lamellae that insert into a midline raphe, thus forming an oval-shaped rosette. In each lamella, there are distributed sensory and nonsensory regions located separately. Three types of receptor cells were identified in the olfactory epithelium: two with cilia or microvilli in the apical border, the third is a different type of sensory cell, the crypt cells with submerged microvilli, and cilia [50,51,52]. The ciliated and microvillous receptor cells are surrounded by supporting cells with small protrusions on their apical surfaces, while the crypt cells are surrounded by one or two specialized electron-lucent supporting cells with microvillous-like apices [51]. The nonsensory epithelium consists of goblet cells, ciliated nonsensory cells and epidermal cells with microridges [51,52,53]. In the olfactory rosette of adult zebrafish, *asic2* mRNA and protein were observed. *asic2* hybridization was observed in the luminal pole of the nonsensory epithelium, particularly, in the cilia basal bodies and ASIC2 immunoreactivity was found only in the cilia of the nonsensory cells. The immunoreaction for ASIC2 was never observed in the olfactory cells [54]. 

### 2.4. Retina

The zebrafish retina consists of the ganglion cell layer (GCL), inner plexiform layer (IPL), inner nuclear layer (INL), outer plexiform layer (OPL), and outer nuclear layer (ONL) [55]. Seven different cell types such as Müller cells and retinal ganglion cells (RGCs), amacrine cells (ACs), horizontal cells (HCs), bipolar cells (BCs), cones and rods are recognized in the zebrafish retina [55,56]. The structural similarities, the genome organization, the regulatory pathways controlling signal transduction and retinal development are highly conserved between zebrafish and humans [56,57]. For these reasons, the zebrafish visual system represents a powerful research tool and provides an excellent animal model to study retinal function and pathology [56,57]. 

In zebrafish, similarly to mammals, *zasic1* mRNA was found in the whole eye of the adults [55]. The specific antibody against ASIC1 recognized, by Western blotting, a band with an estimated molecular weight of about 60 kDa, consistent with the estimated molecular mass of zASIC proteins in zebrafish. The expression of *asic1* in zebrafish retina was analyzed using whole mount in situ hybridization on zebrafish larvae and immunostaining was detected on retina slices [55]. The *zasic1* transcript was detected in 4–5 day post-hatch zebrafish larvae [55]. The immunostaining showed that the ASIC1 immunoreactivity was widely detected in the zebrafish retinal layers, the retinal ganglion cells layer, the inner plexiform layer, the inner nuclear layer, the outer plexiform layer, and the cone photoreceptor layer [55]. In adult zebrafish, mRNA encoding ASIC2 and ASIC4.2 but not zASIC4.1 was detected [33]. ASIC2 was expressed in the outer nuclear layer, the outer plexiform layer, the inner plexiform layer, the ganglion cell layer of the retina, and the optic nerve [33]. ASIC4 was found in the cone and rods layer and the retinal ganglion cell layer. It was also shown that the expression of *asic2* and *asic4.2* was affected by light and darkness [33]. 

## 3. ASICs in Zebrafish Gills

The gills of teleosts are composed of gill arches that provide support for the primary lamellae considered the functional unit of the gill, and are interspersed with five gill slits called chambers [58]. Their epithelium contains ion regulatory cells and, in addition, provides support for the secondary lamellae. The secondary lamellae represent the respiratory unit of the gill and extend from both sides and perpendicular to the longitudinal axis of the main filament [58]. The respiratory lamellae are formed by two epithelial layers separated by spaces due to the presence of pillar cells, through which blood circulates [58]. Freshwater fish, including teleosts, compensate for the loss of ions in a hypotonic environment through the uptake of Na^+^, Cl^−^, and Ca^2+^ ions, which occurs through the activity of particular gill cells, specialized ionocytes defined as mitochondria-rich cells. Four types of ionocytes called mitochondria-rich cells (MRCs) involved in Na^+^ uptake have been demonstrated in zebrafish gills: VHA-rich cells (HR cells), NKA-rich cells (NaR cells), cells expressing Na^+^/Cl^−^ cotransporter (NCC cells), and K^+^-secreting cells (KS cells) [26]. Furthermore, a previous study has shown the involvement of skin keratinocytes in hypotonicity perception and the contribution of the aforenamed in the activation of innate immunity at an early developmental stage of zebrafish embryos through a transient potential receptor vanilloid 4 (TRPV4)/Ca^2+^/TGF-b–activated kinase 1 (TAK1)/NF-kB, by means of pharmacological and genetic inhibition experiments [59]. The expression pattern of different ASIC subunit mRNAs in zebrafish gill tissue was demonstrated by RT-PCR technique. All six ASIC mRNAs were observed in zebrafish gills. Expression of ASIC4.2, by immunoprecipitation, also identified a single band corresponding to ~65 kDa [26]. Furthermore, by immunohistochemistry, cells positive for anti-ASIC4.2 were found in the gills and interlamellar region of the gills. To clarify whether ASIC4.2 colocalizes with NaR cells or HR cells, the gills were double labelled with ASIC4.2 and VHA or NKA, a marker for HR and NaR-type MRC, respectively. Cells immunoreactive for anti-ASIC4.2 were also immunoreactive for anti-VHA. Finally, pharmacological studies in which specific ASIC inhibitors blocked Na^+^ uptake demonstrated the role of ASIC4.2 in regulating Na^+^ uptake in zebrafish exposed to low and ultra-low-sodium media at gill level [26].

## 4. ASICs in Zebrafish Gut 

As in mammals, the enteric nervous system of adult zebrafish is organized into two plexuses, the myenteric and submucosal. Structurally, the myenteric plexus consists mainly of enteric neurons, while the submucous plexus has few neurons and numerous nerve fibers [60]. Specific ASIC2 immunoreactivity was found in the enteric nervous system of adult zebrafish as well as in scattered populations of enteroendocrine cells. Particularly, a subpopulation of neurons and nerve fibers were positive to ASIC2, mainly in the myenteric plexus and occasionally in the submucous one. Immunoreactivity for ASIC2 was also found in enteroendocrine epithelial cells in the gut wall. Most of these ASIC2 immunoreactive cells showed a central soma and two processes directed to the organ lumen and the submucous layer, where sometimes they were found close to ASIC2-positive nerve-fiber profiles [61].

## 5. ASICS in Zebrafish Brain 

ASICs are widely expressed in the nervous system of zebrafish embryos and larvae. The expression of six *zasic* genes in zebrafish neurons was demonstrated through the in situ hybridization technique [30]. In general, the distribution patterns of *zasic* genes were different. Particularly, *zasic1.1* was demonstrated within 30 h postfertilization (hpf) at anterior and posterior lateral line ganglia and optic sensory neurons and at 48 hpf, also in the trigeminal ganglia [30]. At 72 and 96 hpf, expression was observable throughout the central nervous system except for the eyes [30]. *zasic1.2* was evident, although with weak expression at 48 hpf, in the ventral thalamus, ventral midbrain, ventral cerebellum and in the dorsal thalamus, hypothalamus and telencephalon along the anterior commissure. *zasic1. 2* at 48 hpf was present in the dorsal midbrain (dMb) and olfactory bulb, whereas from 96 hpf it was also expressed in the tectum. *zasic1.3* was expressed at 30 hpf in the ganglia of the lateral line while between 30 and 72 hpf in the telencephalon it was expressed along the anterior commissure tract, in the ventral thalamus, ventral midbrain, and ventral cerebellum [30]. By 48 hpf, expression was also evident in the dorsal thalamus and hypothalamus. At 96 hpf, expression was also strong in the habenula. *zasic2* was observed at 30 hpf along the anterior commissure tract and at 48 hpf, also in the preoptic area, ventral thalamus, and ventral midbrain. At 72 and 96 hpf, it was present in the whole brain except for the dorsal forebrain and was also expressed in retinal ganglion cells. *zasic4.1* at 48 hpf showed a similar pattern to that of *zasic1.2*. *zasic4.1* was expressed in the dorsal midbrain and retinal ganglion cells. *zasic4.2* as early as 24 and 30 hpf was expressed along the anterior commissure tract and in cells along the commissure tract. At 48 hpf, it was observable in the preoptic zone, posterior hypothalamus, ventral midbrain, cerebellum, and retinal ganglion cells [30]. 

## 6. Discussion 

The presence of ASICs has been demonstrated in different organs of zebrafish, such as sensory organs, gills, gut, and brain. The expression of ASICs in the hair cells of LLS neuromasts and the neurosensory cells of the inner ear could support the hypothesis of an involvement in the transduction of mechanical stimuli from the aquatic environment similar to the role they play in mammalian inner ear cells. It is known that, functionally, ASICs control moderate deviations from the physiological values of extracellular pH. In the rat and mouse, ASIC1a and ASIC2a immunoreactivity was detected in small vestibular ganglion neurons and afferent fibers of the macula utricle and crista stroma, whereas ASIC2b, ASIC3, and ASIC4 were observed in vestibular ganglion neurons. It has also been demonstrated in these species that the acidification of the extracellular pH generates action potentials in vestibular neurons, confirming a function of ASICs in their excitability [62,63,64]. Regarding taste buds, conflicting data exist in mammals, i.e., ASIC2 has been proposed as a receptor for acidic taste although, in mice lacking the *asic2* gene, behavioral responses to acidic taste stimuli remained unaffected [65,66,67]. There are significant differences in the expression and distribution of *asics* between mammals and zebrafish. Indeed, while *asic4* was demonstrated in zebrafish, it was always absent in mouse and rat, *asic1* and *asic3* have always been absent in zebrafish while they were observed as RNAs in mice and rats. *asic2* was observed in zebrafish and rats but not in mice [67,68]. These differences may be attributable to environmental and dietary differences between mammals and fish. However, the expression of *asic2* and *asic4* in taste buds would also suggest in zebrafish a role as a receptor for acid taste or an involvement of ASICs in taste cells, taste or more functions in zebrafish.

The localization of ASIC2 in the nonsensory epithelium of the olfactory rosette suggests that it is not involved in olfaction. However, the presence of immunoreactivity for ASIC2 in the basal bodies of the cilia of nonsensory cells, notoriously involved in the perception and transduction of mechanical and chemical stimuli, would suggest a role for this ion channel in other types of stimuli from the aquatic environment, pH changes, or movement of water. The presence of *asic1a*, *asic2*, and *asic3* in the mammalian retina has been demonstrated [69,70]. Among the three aforementioned, *asic1* is the most represented and is widely expressed in the retina and involved in retinal activity, particularly in cone function [71]. In mammals, the retina reacts to changes in local pH, presumably to control its acid/base environment in response to systemic acidosis with increased expression, also, of ASICs. The expression of ASICs in the developing and adult retina of zebrafish suggests that they participate, as in mammals, in the mechanism of vision [55,70]. As pH changes have been shown to be associated with pathological conditions, ASICs could also be involved in the pathogenesis of retinal disorders [72,73]. Moreover, it has also been shown that substances blocking the activity of ASIC may represent potential therapeutics in degenerative diseases, like optic nerve degeneration or ischemic retinal diseases [74,75]. Finally, in zebrafish as in mammals, *asic1a*, functionally expressed in retinal pigment epithelium (RPE) cells, might play an important role in the neuroprotection of RPE cells from oxidative stress [73]. The presence of *asics* in zebrafish gills, at mRNA and protein levels, was demonstrated, and their involvement in Na^+^ regulation of adult zebrafish acclimated to low-Na^+^ medium was validated using pharmacological blockade of Na^+^ uptake. Moreover, the presence of alternative transport mechanisms was also hypothesized. The differences found between zebrafish larvae and adults in response to specific inhibitors of ASICs, could be explained by a difference in the ionic compositions and pH used in the two different studies or could suggest the use of different Na^+^ uptake mechanisms in the two stages examined, as previously demonstrated in rainbow trout [25]. Numerous data have demonstrated ASICs in gastrointestinal DRG afferent neurons in mammalian myenteric and submucosal plexus neurons [76]. Indeed, these ion channels would appear to be involved in gastrointestinal physiology [13,16] in the regulation of acid secretion, motility, and mucosal protection in mammals [13,16,77,78]. Increased levels of *asic3* have been observed in gastrointestinal disorders associated with inflammation, while disruption of the *asic2* gene results in altered emptying from the gastrointestinal tract [22]. The role of ASIC2 in the zebrafish gut could be comparable to that observed in mammals, and thus they could be involved in the neuronal responses to acid challenge in the intestinal mucosa, or the tension of the intestinal walls [13,16]. On the other hand, the enteroendocrine cells that expressed positivity for ASIC2 might also be considered chemoreceptors and involved in the regulation of intestinal secretion, releasing intestinal hormones, and/or in maintaining intestinal wall tension. Finally, in the mammalian brain, ASICs are widely expressed in CNS neurons and microglia and play key roles in physiological activities such as synaptic plasticity, microglia Ca^+^ physiology, learning/memory, fear conditioning, and in pathological conditions such as brain ischemia, multiple sclerosis, epileptic seizures, depression-related behavior, anxiety disorders and malignant glioma [79]. In zebrafish, the different *zasic* genes were mainly expressed in the nervous system of embryos and larva, although the expression pattern of the six *zasic* genes, compared to mammals, was limited primarily to neurons and perhaps some glial cells. However, further studies are also needed in the adult zebrafish brain to demonstrate the exact role of ASICs in this experimental model. Therefore, such evidence on the involvement of these ion channels in both physiological and pathological processes in the central nervous system could suggest them as novel and effective therapeutic interventions for CNS diseases.

## 7. Conclusions

In zebrafish the pattern of the distribution of ASICs in different organs and tissues suggests their involvement in multiple physiological functions, including mechanosensation, hearing, temperature sensing, taste, vision, intestinal physiology and brain activity as previously demonstrated in mammals [80,81,82]. From the analysis of these data we can conclude that ASICs are essential for the proper functioning of various organs in zebrafish, and that the latter represents an important experimental model for the study of diseases related to alterations of these ion channels.

## Figures and Tables

**Table 1:** Presence and localization of ASICs and ZASICs in adult and larvae zebrafish with techniques of molecular biology and immunohistochemistry.

	ASIC1	ASIC2	ASIC3	ASIC4	ZASIC1	ZASIC2	ZASIC3	ZASIC4
Neuromast	+	+	+	+	-	-	-	-
Inner ear	+	+	+	-	-	-	-	-
Taste buds	+	+	-	+	-	-	-	-
Olfactory epithelium	-	+	-	-	-	-	-	-
Retina	+	+	-	+	+	-	-	-
Gills	-	-	-	+	-	-	-	-
Intestine	-	+	-	-	-	-	-	-
Brain	-	-	-	-	+	+	-	+

## Data Availability

Data sharing not applicable.
